# Magnesium depletion scores as a risk factor for prevalence and mortality rates of urinary incontinence: a national survey analysis

**DOI:** 10.3389/fnut.2025.1439134

**Published:** 2025-04-30

**Authors:** Ping Xia, Xiaolong Shi, Yunling Yang, Yanru Zhang, Xuyang Hu, Rong Lin, Xiaoying Weng, Fenfang Shen, Xiaobao Chen, Liang Lin

**Affiliations:** ^1^Medical Centre of Maternity and Child Health, Shengli Clinical Medical College of Fujian Medical University, Fuzhou University Affiliated Provincial Hospital, Fuzhou, China; ^2^Department of Urology, Fujian Medical University Union Hospital, Fuzhou, China

**Keywords:** urinary incontinence, stress urinary incontinence, urgent urinary incontinence, NHANES, magnesium

## Abstract

**Background:**

Magnesium regulates vascular smooth muscle contraction, with implications for cardiovascular diseases. However, the population-level relevance of magnesium homeostasis to urinary incontinence (UI) subtypes and associated mortality remains unexamined.

**Methods:**

The National Health and Nutrition Examination Survey (NHANES) were utilized to investigate the association between magnesium depletion score (MDS) and urinary incontinence (UI) from 2005 to 2018. Weighted multivariate regression analyses and multivariate Cox regression analyses were used to analysis. Additionally, subgroup analyses and multiple imputations (MI) were carried out as sensitivity analyses to ensure the strength and reliability of the findings.

**Results:**

A total of 16,197 individuals were included in the study, with 6,881 of them experiencing urinary incontinence (UI). Among those with UI, 767 cases of all-cause mortality were documented. The prevalence rates were 42.83% for stress urinary incontinence (SUI), 27.85% for urgency urinary incontinence (UUI), and 16.82% for mixed urinary incontinence (MUI). Results from weighted logistic regression analysis demonstrated a positive relationship between MDS and SUI (OR 1.09, 95% CI: 1.01–1.17), UUI (OR 1.14, 95% CI: 1.06–1.22), and MUI (OR 1.22, 95% CI: 1.11–1.35). Additionally, higher MDS values were associated with increased severity of urinary incontinence. These findings were further supported by various sensitivity analyses. Furthermore, survey-weighted Cox proportional hazards regression indicated a positive association between MDS and all-cause mortality regardless of (OR 1.27, 95% CI: 1.13–1.41), suggesting that higher MDS independently predicts worse prognosis.

**Conclusion:**

MDS is an important risk factor for the prevalence and mortality rates of UI. Monitoring magnesium status may inform UI prevention strategies. Interpretation should consider limitations including observational design and lack of serum magnesium levels.

## Introduction

Urinary incontinence (UI) is a prevalent condition characterized by the International Continence Society as the involuntary leakage of urine. It is commonly classified into stress urinary incontinence (SUI), urgency urinary incontinence (UUI), and mixed urinary incontinence (MUI) (1, 2). Studies (3–5) suggest that UI affects approximately 25–50% of adult women, with a higher incidence among older individuals. Despite its commonality, less than a quarter of women with UI seek medical intervention (6, 7). The condition can lead to negative psychological effects (8–11) such as depression and anxiety, along with physical discomforts like body odor, sleep disturbances, skin irritation, and an increased risk of falls. This not only impacts individual well-being but also places a substantial economic strain on healthcare systems globally. The development of UI is influenced by various factors (12–17) including chronic illnesses, medication use, childbirth complications, and environmental triggers. Nonetheless, the underlying mechanisms of UI are still unclear.

Given magnesium’s critical role in muscle regulation and nerve function, its deficiency may contribute to the pathophysiology of UI. Emerging evidence suggests that magnesium deficiency may contribute to UI through its role in smooth muscle regulation. Magnesium acts as a natural calcium antagonist, inhibiting voltage-gated calcium channels and reducing detrusor muscle overactivity (18). Clinical trials have shown that magnesium supplementation alleviates postoperative bladder discomfort (19–21) and improves urgency symptoms in women with detrusor instability (22). These findings suggest a potential mechanistic link between magnesium status and bladder dysfunction. Magnesium is a crucial trace element in the human body, playing various essential roles. It acts as a cofactor for multiple enzymes, regulating their functions, and is involved in the regulation of ion channels, modulation of the neurosystem, cardiovascular functions, and other important processes (23–25). In clinical settings, serum magnesium concentration is often utilized to diagnose systemic magnesium deficiency. However, as serum magnesium represents only a small portion of the total body magnesium, with the majority stored in bones and tissues, blood tests may not provide a complete assessment of an individual’s magnesium status (26). Current research (27) indicates that serum magnesium is not the best predictor for diagnosing magnesium deficiency in the body. Alternative methods, such as 24-h urinary magnesium excretion, offer a more accurate evaluation of total body magnesium content. The magnesium tolerance test (MTT) is considered the most reliable method, although its complexity and the need for pre- and post-intravenous magnesium injection urine samples limit its clinical utility. The MDS was first established and psychometrically evaluated within the Personalized Prevention of Colorectal Cancer Trial, utilizing a cohort of 77 participants demonstrating elevated susceptibility to magnesium depletion. It demonstrated strong correlation with magnesium deficiency confirmed by the MTT and showed utility in predicting systemic inflammation and cardiovascular mortality, thereby supporting its reliability as a clinical tool for assessing magnesium status (28). Although magnesium’s role in cardiovascular and metabolic diseases is well-established (23, 29–36), its association with urinary incontinence (UI) remains underexplored. Prior research (26, 27) focused on serum magnesium levels, which may not fully reflect systemic magnesium status. To our knowledge, no study has evaluated the MDS in relation to UI risk or prognosis.

Limited research exists on the association between magnesium and urinary incontinence. This study aimed to investigate the association of MDS on UI and its prognostic relevance. We hypothesized that higher MDS scores would be associated with an increased risk of UI and poorer prognostic outcomes. The National Health and Nutrition Examination Survey data was utilized for cross-sectional and cohort analyses to explore the relationship between MDS and UI.

## Materials and methods

### Study population

NHANES is a national initiative led by the National Center for Health Statistics (NCHS) to evaluate nutritional status and its influence on health and disease prevention. The survey involves interviews and physical examinations conducted by trained healthcare professionals. This study utilized cross-sectional and cohort study designs, analyzing data from NHANES between 2005 and 2018. Data on Complete UI and MDS were collected from participants. NHANES was approved by the Centers for Disease Control and Prevention (CDC) National Center for Research Ethics in Health Statistics, and written informed consent was obtained from all adult participants.

### Assessment of UI

UI is characterized and categorized through two interviews within the “Kidney Conditions-Urology” section. The topic of urinary incontinence was specifically discussed with participants aged 20 and older. If the participant answers “yes” to the question “During the past 12 months, have you leaked or lost control of even a small amount of urine with an activity like coughing, lifting, or exercise?,” it indicates SUI. If the participant answers “yes” to the question “During the past 12 months, have you leaked or lost control of even a small amount of urine with an urge or pressure to urinate and you could not get to the toilet fast enough?,” it indicates UUI (37, 38). Participants who answered affirmatively to both questions were classified as having MUI. Both questions aimed to determine the frequency of these incidents (daily, several times per week, several times per month, several times per year) and the severity of urinary leakage each time (drops, splashes, or more).

All eligible participants with sufficient identifying information were linked to mortality data using the National Death Index from the National Center for Health Statistics Research Data. The association was established through publicly available linked mortality files from the National Center for Health Statistics, with a focus on deaths from any cause. The follow-up period began at the time of completion of the NHANES questionnaire and ended at the time of death or December 31, 2019.

### Assessment of MDS

The MDS is determined based on specific criteria (28), including the current use of diuretics (1 point), proton pump inhibitors (1 point), and excessive alcohol consumption (1 point), as well as the classification of renal function. Renal function is classified into normal (score 0), mild deterioration (score 1), and severe deterioration (score 2) based on glomerular filtration rate (eGFR) calculated using the CKD-EPI formula. Participants were categorized into normal renal function [eGFR ≥ 90 mL/(min 1.73 m^2^)], mildly impaired renal function [60 mL/(min 1.73 m^2^) ≤ eGFR < 90 mL/(min 1.73 m^2^)], and severely impaired renal function [eGFR < 60 mL/(min 1.73 m^2^)].

### Covariates

The variables examined in this study included demographic characteristics such as age, body mass index, marital status, education, race, and household income. Lifestyle habits like physical activity and smoking, comorbidities such as coronary heart disease, hypertension, metabolic syndrome, diabetes, previous cancer, and hysterectomy were also considered. Additionally, dietary magnesium intake and total energy intake were included in the analysis. The diagnostic criteria for diabetes comprised glycosylated hemoglobin (HbA1c) ≥6.5%, fasting plasma glucose ≥126 mg/dL, use of antidiabetic medications, or self-report. Hypertension diagnosis was based on systolic/diastolic blood pressure ≥140/90 mmHg, use of antihypertensive medication, or self-report. Cardiovascular disease diagnosis included coronary heart disease, congestive heart failure, heart attack, stroke, and angina. Metabolic syndrome in adults was determined according to the National Cholesterol Education Program Adult Treatment Group III criteria (39, 40). The Systemic Immune-Inflammation Index (SII) (41) was calculated using the formula: SII = [Neutrophil count (10^9^/L)] × [Platelet count (10^9^/L)]/[Lymphocyte count (10^9^/L)]. Data on dietary magnesium intake (mg/day) and energy intake were collected through two 24-h dietary recall interviews, encompassing intake from both food and beverages. Initial data collection was at the Mobile Examination Center (MEC), followed by a second telephone survey 3 to 10 days later. To ensure result objectivity, average magnesium intake and total energy intake for each participant were calculated based on data from both time points.

### Statistical analysis

The data processing in our study adhered to the NHANES analysis guidelines. None of the variables had missing data exceeding 10%. All analyses were performed using appropriate sampling weights. Weighted means (standard errors) were utilized to present continuous data, whereas weighted percentages (standard errors) were employed for categorical data. Student’s *t*-test was used to compare baseline characteristics for differences in continuous data, while the chi-square test was used for categorical data.

Weighted multiple logistic regression analysis was used to investigate the association between MDS and UI, while adjusting for various demographic and health factors (age, race, marital status, education, PIR, BMI, moderate and vigorous activity, smoking status, DM, CVD, hypertension, cancer, MetS, hyperlipidemia, vaginal delivery, hysterectomy, albumin, SII, magnesium intake, energy, creatinine, and uric acid). Additionally, weighted multivariable Cox proportional hazards regression analysis was conducted to assess the association between MDS and all-cause mortality risk. Similar adjustments were made in both analyses. The comparison of cumulative survival rates across different MDS categories was plotted using weighted Kaplan–Meier curves with log-rank tests. Multiple sensitivity analyses were performed, including categorizing MDS as a variable, subgroup analyses and multiple imputation. All statistical analyses were conducted using R software (version 4.2.0) and Free Statistics software (version 1.9.2).

## Results

### Participant characteristics

**Figure 1:**
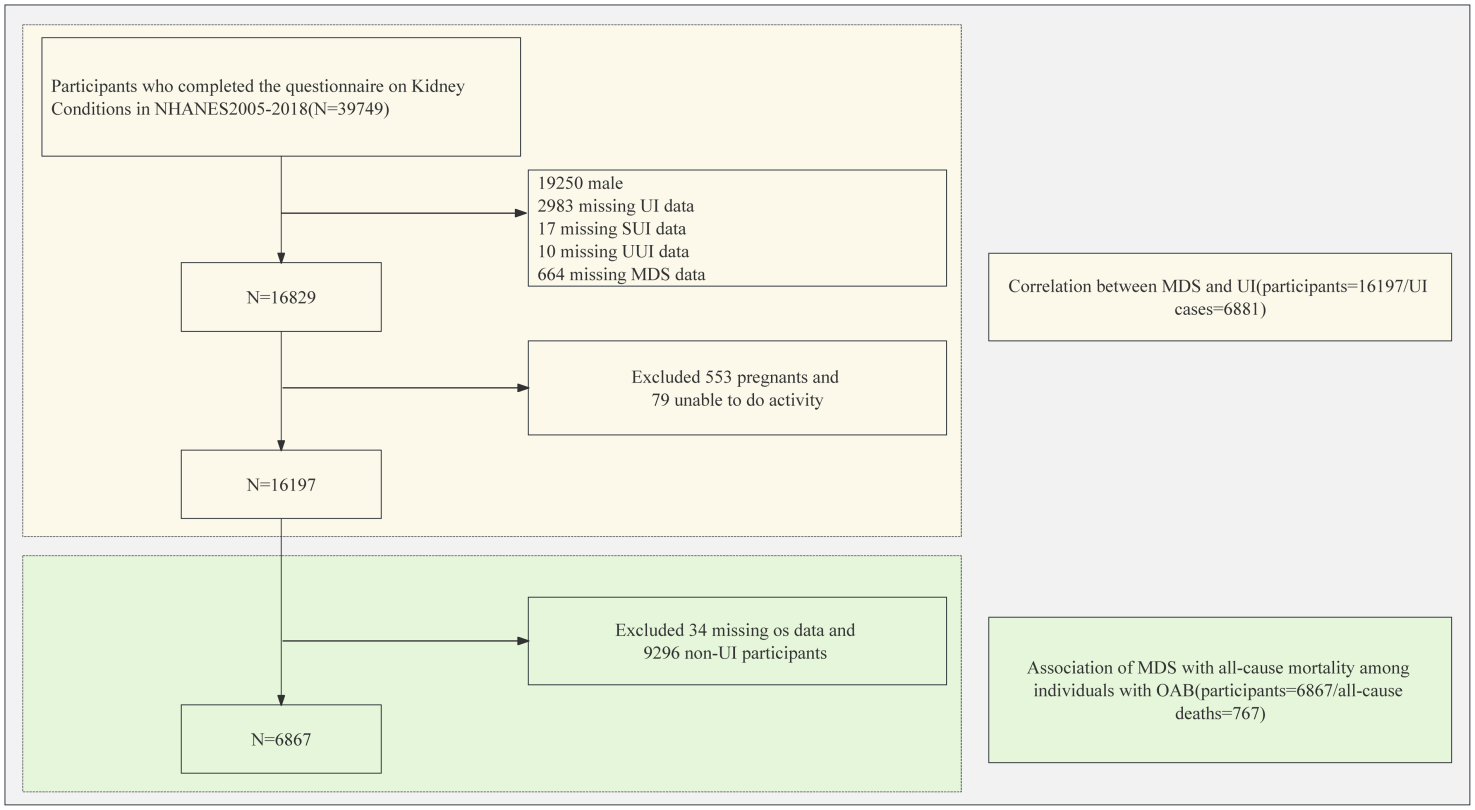
Flowchart of the sample selection from the National Health and Nutrition Examination Survey 2005 to 2018. UI, urinary incontinence; MDS, magnesium depletion score; NHANES, National Health and Nutrition Examination Survey.

**Figure 2:**
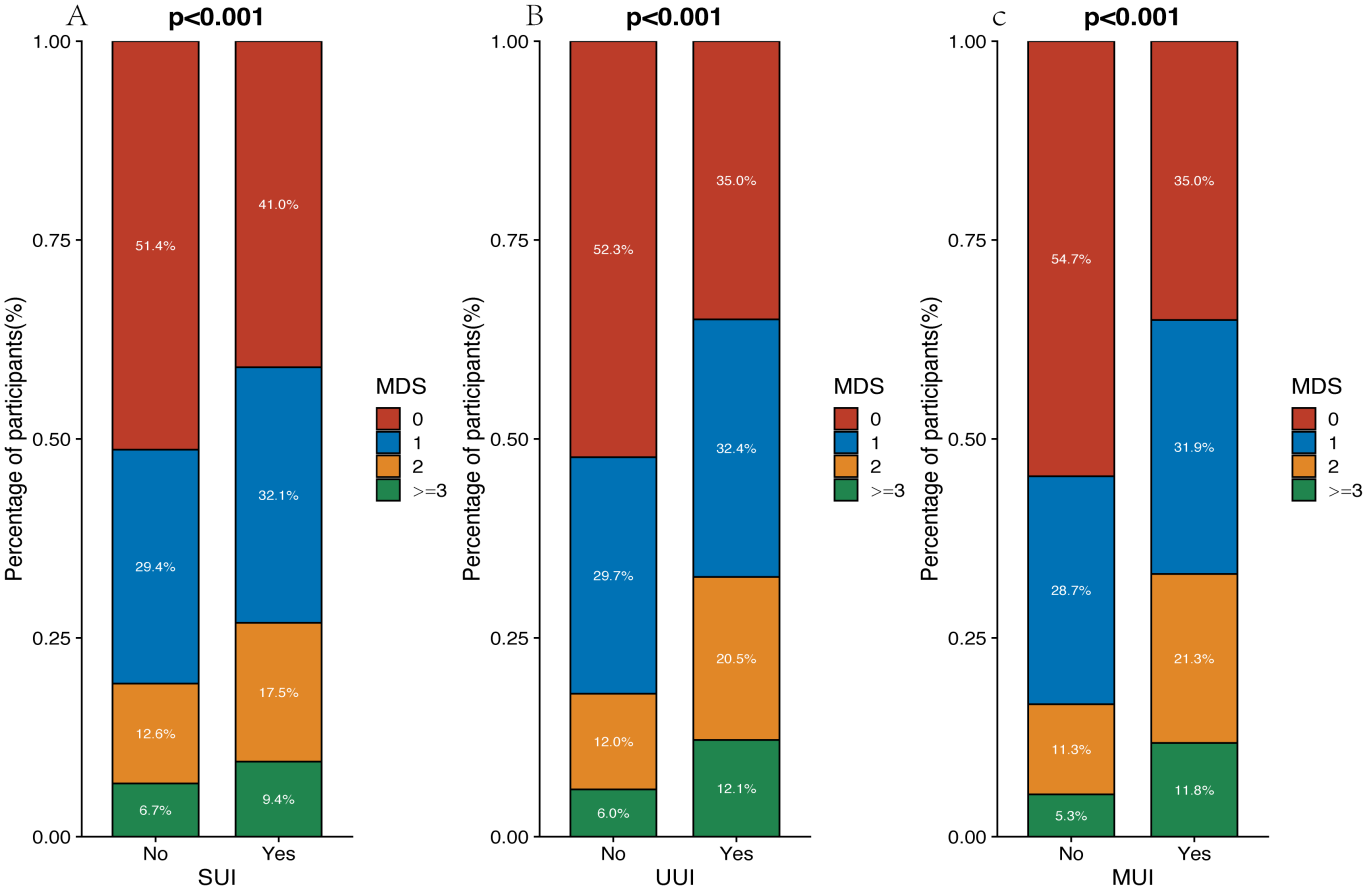
The distribution of MDS in UI, SUI **(A)**, UUI **(B)**, and MUI **(C)**.

Table 1 presents the demographic characteristics of the participants. Following the screening process, 16,197 cases met the predefined criteria as shown in Figure 1. The average age of the participants was 48.57 years, with prevalence rates of SUI at 42.83%, UUI at 27.85%, and MUI at 16.82%. Figure 2 illustrates the distribution of different MDS levels among individuals with SUI, UUI, and MUI. The data shows that participants with UI have a higher proportion in the group where MDS >0 compared to those without UI. It is worth noting that individuals with higher MDS demonstrated higher age, BMI, a greater history of hysterectomy and vaginal delivery, increased comorbidities (such as diabetes, cardiovascular disease, hypertension, tumors, metabolic syndrome, and hyperlipidemia), higher SII, as well as elevated levels of creatinine and uric acid.

**Table 1:** Demographic and clinical parameters according to MDS (*N* = 16,197).

Variable	Total	MDS = 0	MDS = 1	MDS = 2	MDS ≥ 3	*p*-value
Age (years)	48.57 ± 0.26	39.28 ± 0.24	50.98 ± 0.34	61.37 ± 0.39	68.68 ± 0.38	<0.0001
Age, *n* (%)						<0.0001
<50 years	52.54 (0.01)	76.05 (0.77)	45.95 (1.07)	19.77 (1.28)	4.68 (0.69)	
≥50 years	47.46 (0.01)	23.95 (0.77)	54.05 (1.07)	80.23 (1.28)	95.32 (0.69)	
BMI, *n* (%)						<0.0001
<25 kg/m^2^	33.08 (0.01)	35.56 (0.91)	35.54 (0.99)	27.90 (1.31)	19.69 (1.36)	
25–30 kg/m^2^	27.68 (0.01)	26.88 (0.73)	27.76 (0.87)	29.56 (1.14)	30.92 (1.70)	
≥30 kg/m^2^	38.59 (0.01)	37.56 (0.80)	36.70 (0.98)	42.54 (1.39)	49.39 (1.78)	
Race, *n* (%)						<0.0001
Non-Hispanic White	68.61 (0.03)	56.99 (1.49)	75.57 (1.22)	81.42 (1.19)	81.81 (1.39)	
Non-Hispanic Black	11.62 (0.01)	13.82 (0.84)	9.91 (0.71)	9.15 (0.84)	11.11 (1.06)	
Mexican American	7.44 (0.01)	11.90 (0.93)	4.52 (0.44)	3.23 (0.47)	2.08 (0.41)	
Other race	12.32 (0.01)	17.29 (0.81)	9.99 (0.63)	6.20 (0.56)	5.00 (0.64)	
Marital status, *n* (%)						<0.0001
Solitude	40.07 (0.01)	38.14 (0.89)	38.95 (1.02)	43.84 (1.36)	49.84 (2.16)	
Cohabitation	59.88 (0.02)	61.86 (0.89)	61.05 (1.02)	56.16 (1.36)	50.16 (2.16)	
Education level, *n* (%)						<0.0001
Less than or high school	37.16 (0.01)	36.97 (0.96)	34.84 (1.15)	38.46 (1.26)	46.87 (1.86)	
Above high school	62.80 (0.02)	63.03 (0.96)	65.16 (1.15)	61.54 (1.26)	53.13 (1.86)	
PIR, *n* (%)						<0.0001
<1.3	20.48 (0.01)	25.63 (0.80)	18.38 (0.81)	18.27 (1.02)	22.68 (1.50)	
1.3–3.5	33.99 (0.01)	36.74 (0.82)	34.18 (1.01)	37.12 (1.30)	42.55 (1.98)	
≥3.5	39.05 (0.01)	37.62 (1.15)	47.44 (1.28)	44.61 (1.57)	34.77 (2.18)	
Smoking status, *n* (%)						<0.0001
Never	61.21 (0.01)	65.76 (0.83)	58.29 (1.00)	56.53 (1.45)	56.60 (1.68)	
Former	20.93 (0.01)	15.26 (0.66)	22.43 (0.86)	28.72 (1.41)	33.21 (1.64)	
Current	17.83 (0.01)	18.98 (0.69)	19.28 (0.85)	14.75 (0.87)	10.19 (1.08)	
Moderate, *n* (%)						<0.0001
No	51.89 (0.01)	51.62 (0.81)	48.78 (1.05)	54.12 (1.49)	63.74 (2.18)	
Yes	48.10 (0.01)	48.38 (0.81)	51.22 (1.05)	45.88 (1.49)	36.26 (2.18)	
Vigorous, *n* (%)						<0.0001
No	76.81 (0.02)	73.03 (0.78)	75.43 (1.18)	84.78 (1.08)	90.47 (1.35)	
Yes	23.18 (0.01)	26.97 (0.78)	24.57 (1.18)	15.22 (1.08)	9.53 (1.35)	
Hysterectomy, *n* (%)						<0.0001
No	71.43 (0.02)	86.86 (0.69)	74.97 (0.87)	62.83 (1.20)	50.83 (1.97)	
Yes	22.06 (0.01)	13.14 (0.69)	25.03 (0.87)	37.17 (1.20)	49.17 (1.97)	
Vaginal delivery, *n* (%)						<0.0001
0	16.06 (0.01)	23.74 (0.74)	18.90 (0.78)	14.83 (1.21)	12.34 (1.45)	
1–3	38.00 (0.01)	47.99 (0.85)	47.84 (1.04)	45.61 (1.53)	37.40 (1.67)	
≥3	27.28 (0.01)	28.26 (0.76)	33.25 (0.99)	39.56 (1.45)	50.26 (1.78)	
DM, *n* (%)						<0.0001
No	78.63 (0.02)	85.25 (0.49)	79.27 (0.86)	69.29 (1.19)	57.21 (1.70)	
PreDM	7.34 (0.00)	5.87 (0.36)	7.52 (0.57)	9.82 (0.73)	10.85 (1.24)	
DM	13.72 (0.00)	8.88 (0.37)	13.22 (0.65)	20.89 (1.00)	31.94 (1.78)	
CVD, *n* (%)						<0.0001
No	92.24 (0.02)	96.92 (0.26)	93.44 (0.42)	85.41 (0.86)	71.46 (1.77)	
Yes	7.76 (0.00)	3.08 (0.26)	6.56 (0.42)	14.59 (0.86)	28.54 (1.77)	
Hypertension, *n* (%)						<0.0001
No	62.15 (0.02)	79.03 (0.69)	62.51 (1.04)	34.73 (1.31)	11.89 (1.32)	
Yes	37.84 (0.01)	20.97 (0.69)	37.49 (1.04)	65.27 (1.31)	88.11 (1.32)	
Cancer, *n* (%)						<0.0001
No	88.37 (0.02)	93.46 (0.43)	87.49 (0.62)	81.21 (0.98)	76.71 (1.45)	
Yes	11.54 (0.00)	6.54 (0.43)	12.51 (0.62)	18.79 (0.98)	23.29 (1.45)	
MetS, *n* (%)						<0.0001
No	67.60 (0.02)	76.66 (0.68)	68.89 (0.91)	53.60 (1.60)	37.12 (2.06)	
Yes	32.09 (0.01)	23.34 (0.68)	31.11 (0.91)	46.40 (1.60)	62.88 (2.06)	
Hyperlipidemia, *n* (%)						<0.0001
No	30.28 (0.01)	38.69 (0.86)	29.61 (1.02)	16.90 (1.06)	8.57 (0.90)	
Yes	69.72 (0.02)	61.31 (0.86)	70.39 (1.02)	83.10 (1.06)	91.43 (0.90)	
Albumin (g/L)	41.73 ± 0.06	41.75 ± 0.07	41.88 ± 0.07	41.59 ± 0.09	41.26 ± 0.15	<0.0001
SII	566.50 ± 4.34	558.40 ± 4.96	565.57 ± 6.83	571.04 ± 8.52	610.35 ± 13.27	0.002
Dietary magnesium intake (mg)	267.46 ± 1.82	263.86 ± 2.45	280.13 ± 2.69	263.93 ± 2.91	237.26 ± 3.93	<0.0001
Dietary energy (kcal)	1823.83 ± 8.15	1835.64 ± 10.06	1875.01 ± 15.07	1778.63 ± 20.24	1601.31 ± 23.05	<0.0001
Creatinine (μmol/L)	69.73 ± 0.32	60.27 ± 0.18	69.42 ± 0.26	81.87 ± 1.22	101.96 ± 1.43	<0.0001
Uric acid (μmol/L)	287.02 ± 0.84	264.23 ± 0.96	284.15 ± 1.44	317.57 ± 1.99	371.63 ± 3.12	<0.0001
SUI						<0.0001
No	57.17 (0.01)	63.41 (0.75)	54.94 (0.89)	48.50 (1.25)	46.65 (1.88)	
Yes	42.83 (0.01)	36.59 (0.75)	45.06 (0.89)	51.50 (1.25)	53.35 (1.88)	
UUI						<0.0001
No	72.15 (0.02)	79.48 (0.66)	71.31 (0.95)	60.66 (1.38)	54.11 (1.68)	
Yes	27.85 (0.01)	20.52 (0.66)	28.69 (0.95)	39.34 (1.38)	45.89 (1.68)	
MUI						<0.0001
No	53.09 (0.01)	83.75 (0.61)	74.59 (1.05)	61.36 (1.68)	55.77 (2.26)	
Yes	16.82 (0.00)	16.25 (0.61)	25.41 (1.05)	38.64 (1.68)	44.23 (2.26)	

BMI, body mass index; PIR, poverty to income ratio; DM, diabetes mellitus; CVD, cardiovascular disease; MetS, metabolic syndrome; SII, system immune-inflammation index; SUI, stress urinary incontinence; UUI, urgent urinary incontinence; MUI, mix urinary incontinence; MDS, magnesium depletion scores. Values are mean ± SD (continuous variables) or *n*% (categorical variables) are weighted.

### The association between MDS and UI

The association between MDS and UI was examined through weighted logistic regression analysis (Table 2). In the case of SUI, a significant positive association was observed between the continuous MDS score and SUI without any adjustments (Crude model: OR = 1.29, 95% CI = 1.23–1.35). Upon controlling for covariates in Model 1, Model 2, and Model 3, MDS continued to be positively linked to SUI, albeit with a reduced OR (OR = 1.09, 95% CI = 1.01–1.17). When MDS was categorized with MDS = 0 as the reference, the positive relationship with SUI persisted across all models, with a significant linear trend noted between increasing MDS and heightened SUI risk (*p* < 0.05).

**Table 2:** Association between MDS and UI.

Variables	Unadjusted model	Model 1 (95% CI)	Model 2 (95% CI)	Model 3 (95% CI)
SUI				
MDS continue	1.29 (1.23, 1.35)	1.11 (1.05, 1.18)	1.07 (1.00, 1.14)	1.09 (1.01, 1.17)
MDS category				
MDS = 0	ref	ref	ref	ref
MDS = 1	1.42 (1.30, 1.56)	1.22 (1.09, 1.36)	1.18 (1.05, 1.32)	1.21 (1.06, 1.39)
MDS = 2	1.84 (1.63, 2.08)	1.35 (1.15, 1.59)	1.26 (1.06, 1.49)	1.31 (1.07, 1.60)
MDS ≥ 3	1.98 (1.66, 2.37)	1.29 (1.04, 1.59)	1.13 (0.91, 1.40)	1.20 (0.94, 1.55)
*p* for trend	<0.0001	<0.001	0.02	0.02
UUI				
MDS continue	1.49 (1.42, 1.56)	1.15 (1.09, 1.22)	1.10 (1.04, 1.17)	1.14 (1.06, 1.22)
MDS category				
MDS = 0	ref	ref	ref	ref
MDS = 1	1.56 (1.39, 1.75)	1.20 (1.05, 1.37)	1.17 (1.02, 1.35)	1.22 (1.04, 1.44)
MDS = 2	2.51 (2.17, 2.90)	1.45 (1.21, 1.73)	1.34 (1.12, 1.62)	1.47 (1.19, 1.81)
MDS ≥ 3	3.28 (2.82, 3.82)	1.51 (1.25, 1.82)	1.30 (1.07, 1.59)	1.43 (1.13, 1.81)
*p* for trend	<0.0001	<0.0001	0.001	<0.001
MUI				
MDS continue	1.63 (1.53, 1.73)	1.21 (1.12, 1.30)	1.14 (1.05, 1.23)	1.22 (1.11, 1.35)
MDS category				
MDS = 0	ref	ref	ref	ref
MDS = 1	1.76 (1.52, 2.03)	1.31 (1.10, 1.55)	1.25 (1.05, 1.49)	1.39 (1.14, 1.69)
MDS = 2	3.24 (2.72, 3.87)	1.75 (1.39, 2.20)	1.54 (1.20, 1.97)	1.84 (1.40, 2.42)
MDS ≥ 3	4.09 (3.30, 5.06)	1.63 (1.27, 2.10)	1.33 (1.01, 1.75)	1.66 (1.19, 2.31)
*p* for trend	<0.0001	<0.0001	0.002	<0.0001

Unadjusted model: No covariates were adjusted. Model 1: Age, race, marital status, education, PIR, BMI, moderate and vigorous activity were adjusted. Model 2: Model 1 + smoking status, DM, CVD, hypertension, cancer, MetS, and hyperlipidemia were adjusted. Model 3: Model 2 + vaginal delivery, hysterectomy, albumin, SII, magnesium intake, energy, creatinine, and uric acid were adjusted.

Similarly, the association between MDS and UUI and MUI demonstrated comparable patterns to MDS and SUI. MDS exhibited a positive association with both UUI and MUI, whether analyzed as a continuous or categorical variable. The initial OR diminished after adjustments for various models and confounding variables.

Table 3 revealed a positive association between the severity of urinary incontinence and MDS. When comparing MDS levels to urine leakage per episode, “small splashes” and “more” had unadjusted OR values of 1.2 (95% CI = 1.12–1.28) and 1.58 (95% CI = 1.44–1.72) respectively. After adjusting for different models, the association persisted but with decreased OR values. Similarly, when examining MDS in relation to the frequency of urinary incontinence, “a few times a week” and “every day and/or night” groups displayed significant *p*-values (<0.05), indicating a strong link. However, the “a few times a month” group did not show a significant association. Overall, our findings suggest that as the frequency or volume of urine leakage increases, there is a corresponding rise in OR values, highlighting the positive association between MDS and the severity of urinary incontinence.

**Table 3:** Association between MDS and severity of UI.

Variables	Unadjusted model	Model 1	Model 2	Model 3
UI volume				
Drops	ref	ref	ref	ref
Small splashes	1.20 (1.12, 1.28)	1.14 (1.05, 1.23)	1.11 (1.02, 1.20)	1.10 (0.99, 1.21)
More	1.58 (1.44, 1.72)	1.31 (1.17, 1.45)	1.24 (1.11, 1.39)	1.23 (1.06, 1.42)
UI frequency				
Less than once a month	ref	ref	ref	ref
A few times a month	1.13 (1.04, 1.23)	1.07 (0.97, 1.19)	1.06 (0.96, 1.18)	1.05 (0.93, 1.19)
A few times a week	1.34 (1.23, 1.47)	1.24 (1.12, 1.38)	1.22 (1.10, 1.36)	1.20 (1.05, 1.37)
Every day and/or night	1.68 (1.56, 1.82)	1.30 (1.17, 1.44)	1.20 (1.08, 1.33)	1.27 (1.11, 1.45)

Unadjusted model: No covariates were adjusted. Model 1: Age, race, marital status, education, PIR, BMI, moderate, and vigorous activity were adjusted. Model 2: Model 1 + smoking status, DM, CVD, hypertension, cancer, MetS, and hyperlipidemia were adjusted. Model 3: Model 2 + vaginal delivery, hysterectomy, albumin, SII, magnesium intake, energy, creatinine, and uric acid were adjusted.

### Subgroup analysis

Subgroup analyses were performed to further investigate the relationship between MDS and different types of UI (Table 4). The results indicated that MDS was significantly associated with SUI in subgroups characterized by age <50, delivery = 0, and no hypertension. An interaction effect of age (<50 or ≥50) was observed, suggesting that the impact of MDS on SUI varies across age groups. Similar interaction effects were also noted in the delivery and hypertension subgroups. In the subgroup analysis of BMI ≥25 and magnesium intake Q1 group, MDS showed a significant association with UUI, with an interaction effect observed in the CVD subgroup (*p* for interaction = 0.04), indicating variability in the association of MDS on UUI among different CVD subgroups. For MUI subgroup analysis, MDS exhibited a positive association with MUI in most subgroups, except for the BMI <25 and PIR = 1.3–3.5 group. No significant interaction effects were found among the subgroups (all *p* for interaction >0.05).

**Table 4:** Subgroup analysis of the association between MDS and UI.

Character	SUI		UUI		MUI	
	OR (95% CI)	*p* for interaction	OR (95% CI)	*p* for interaction	OR (95% CI)	*p* for interaction
Age		0.005		0.19		0.24
<50 years	1.43 (1.04, 1.98)		0.94 (0.76, 1.17)		1.23 (1.11, 1.38)	
≥50 years	0.95 (0.84, 1.09)		1.10 (0.96, 1.26)		1.38 (1.12, 1.71)	
BMI		0.03		0.63		0.34
<25 kg/m^2^	1.03 (0.79, 1.35)		1.12 (0.85, 1.47)		1.19 (0.97, 1.44)	
25–30 kg/m^2^	0.93 (0.75, 1.17)		0.81 (0.67, 0.97)		1.36 (1.18, 1.56)	
≥30 kg/m^2^	1.09 (0.91, 1.29)		1.22 (1.04, 1.42)		1.10 (0.89, 1.36)	
Race		0.58		0.49		0.1
Non-Hispanic White	1.06 (0.91, 1.22)		1.06 (0.92, 1.21)		1.21 (0.94, 1.56)	
Non-Hispanic Black	0.99 (0.78, 1.27)		0.96 (0.77, 1.20)		1.00 (0.84, 1.20)	
Mexican American	1.05 (0.74, 1.48)		1.02 (0.73, 1.43)		1.15 (0.92, 1.44)	
Other race	0.80 (0.61, 1.05)		1.41 (0.99, 2.01)		1.24 (1.10, 1.40)	
Marital status		0.2		0.1		0.34
Solitude	1.13 (0.95, 1.35)		0.97 (0.81, 1.16)		1.25 (1.10, 1.42)	
Cohabitation	0.96 (0.82, 1.12)		1.13 (0.97, 1.32)		1.24 (1.09, 1.42)	
Education level		0.08		0.38		0.37
Less than or high school	0.97 (0.80, 1.17)		1.05 (0.88, 1.26)		1.29 (1.11, 1.50)	
Above high school	1.06 (0.91, 1.25)		1.08 (0.92, 1.28)		1.20 (1.06, 1.35)	
PIR		0.31		0.62		0.35
<1.3	1.06 (0.88, 1.28)		1.19 (0.99, 1.43)		1.38 (1.21, 1.58)	
1.3–3.5	0.91 (0.75, 1.11)		1.03 (0.86, 1.24)		1.14 (0.95, 1.35)	
≥3.5	1.13 (0.93, 1.37)		1.07 (0.89, 1.27)		1.27 (1.10, 1.47)	
Smoking status		0.37		0.52		0.61
Never	0.98 (0.83, 1.15)		1.02 (0.86, 1.20)		1.25 (1.06, 1.48)	
Former	1.09 (0.88, 1.34)		1.13 (0.92, 1.38)		1.24 (1.09, 1.42)	
Current	1.21 (0.85, 1.73)		1.06 (0.82, 1.37)		1.31 (1.06, 1.62)	
Moderate		0.59		0.91		0.34
No	0.96 (0.83, 1.12)		1.02 (0.87, 1.19)		1.26 (1.12, 1.41)	
Yes	1.12 (0.92, 1.37)		1.13 (0.94, 1.35)		1.24 (1.06, 1.45)	
Vigorous		0.88		0.14		0.2
No	1.03 (0.91, 1.17)		1.09 (0.96, 1.22)		1.27 (1.14, 1.41)	
Yes	1.03 (0.71, 1.51)		0.93 (0.65, 1.32)		1.05 (0.74, 1.48)	
Hysterectomy		0.71		0.33		0.17
No	1.04 (0.90, 1.19)		1.03 (0.88, 1.21)		1.24 (1.07, 1.42)	
Yes	1.02 (0.84, 1.24)		1.10 (0.91, 1.33)		1.26 (1.11, 1.43)	
Vaginal delivery		0.03		0.97		0.08
0	1.37 (1.00, 1.90)		1.13 (0.85, 1.51)		1.27 (1.12, 1.44)	
1–3	0.92 (0.76, 1.11)		1.06 (0.89, 1.26)		1.31 (1.01, 1.71)	
≥3	1.05 (0.88, 1.26)		1.03 (0.86, 1.25)		1.23 (1.06, 1.42)	
DM		0.24		0.54		0.97
No	1.04 (0.90, 1.19)		1.03 (0.88, 1.20)		1.24 (1.04, 1.49)	
PreDM	0.80 (0.52, 1.25)		1.29 (0.87, 1.91)		1.50 (1.08, 2.08)	
DM	1.00 (0.82, 1.22)		1.03 (0.84, 1.26)		1.20 (1.06, 1.36)	
CVD		0.15		0.04		0.63
No	1.07 (0.94, 1.21)		1.04 (0.91, 1.18)		1.24 (1.11, 1.38)	
Yes	0.80 (0.58, 1.09)		1.29 (0.96, 1.72)		1.30 (1.04, 1.62)	
Hypertension		<0.001		0.54		0.06
No	1.35 (1.04, 1.76)		1.04 (0.83, 1.32)		1.44 (1.22, 1.70)	
Yes	0.92 (0.80, 1.05)		1.08 (0.93, 1.26)		1.16 (1.03, 1.31)	
Cancer		0.44		0.73		0.95
No	1.02 (0.90, 1.17)		1.09 (0.97, 1.23)		1.24 (1.12, 1.39)	
Yes	1.02 (0.76, 1.38)		0.86 (0.62, 1.18)		1.25 (1.01, 1.54)	
MetS		0.13		0.3		0.17
No	1.11 (0.94, 1.31)		1.01 (0.85, 1.20)		1.23 (1.08, 1.40)	
Yes	0.95 (0.80, 1.12)		1.12 (0.96, 1.29)		1.26 (1.08, 1.46)	
Hyperlipidemia		0.68		0.44		0.05
No	1.08 (0.80, 1.45)		1.02 (0.77, 1.35)		1.45 (1.16, 1.81)	
Yes	1.03 (0.91, 1.17)		1.06 (0.93, 1.21)		1.21 (1.09, 1.35)	
Dietary magnesium intake (mg)		0.17		0.22		0.06
Q1	1.12 (0.88, 1.44)		1.31 (1.02, 1.68)		1.48 (1.23, 1.78)	
Q2	0.95 (0.78, 1.15)		1.14 (0.94, 1.38)		1.07 (0.92, 1.26)	
Q3	1.08 (0.84, 1.38)		1.01 (0.80, 1.28)		1.42 (1.15, 1.75)	
Q4	0.87 (0.68, 1.12)		0.91 (0.70, 1.18)		1.08 (0.89, 1.31)	

### Sensitivity analysis

Multiple imputation techniques were utilized to address missing values, resulting in the creation of five datasets for analysis. Logistic regression analysis was conducted to explore the relationship between imputed MDS and UI. The adjusted models remained consistent with the covariates adjusted in the previous Model 3. Findings from Supplementary Table 2 revealed a positive association between MDS and UI across SUI, UUI, and MUI subtypes.

### The association between MDS and mortality

**Figure 3:**
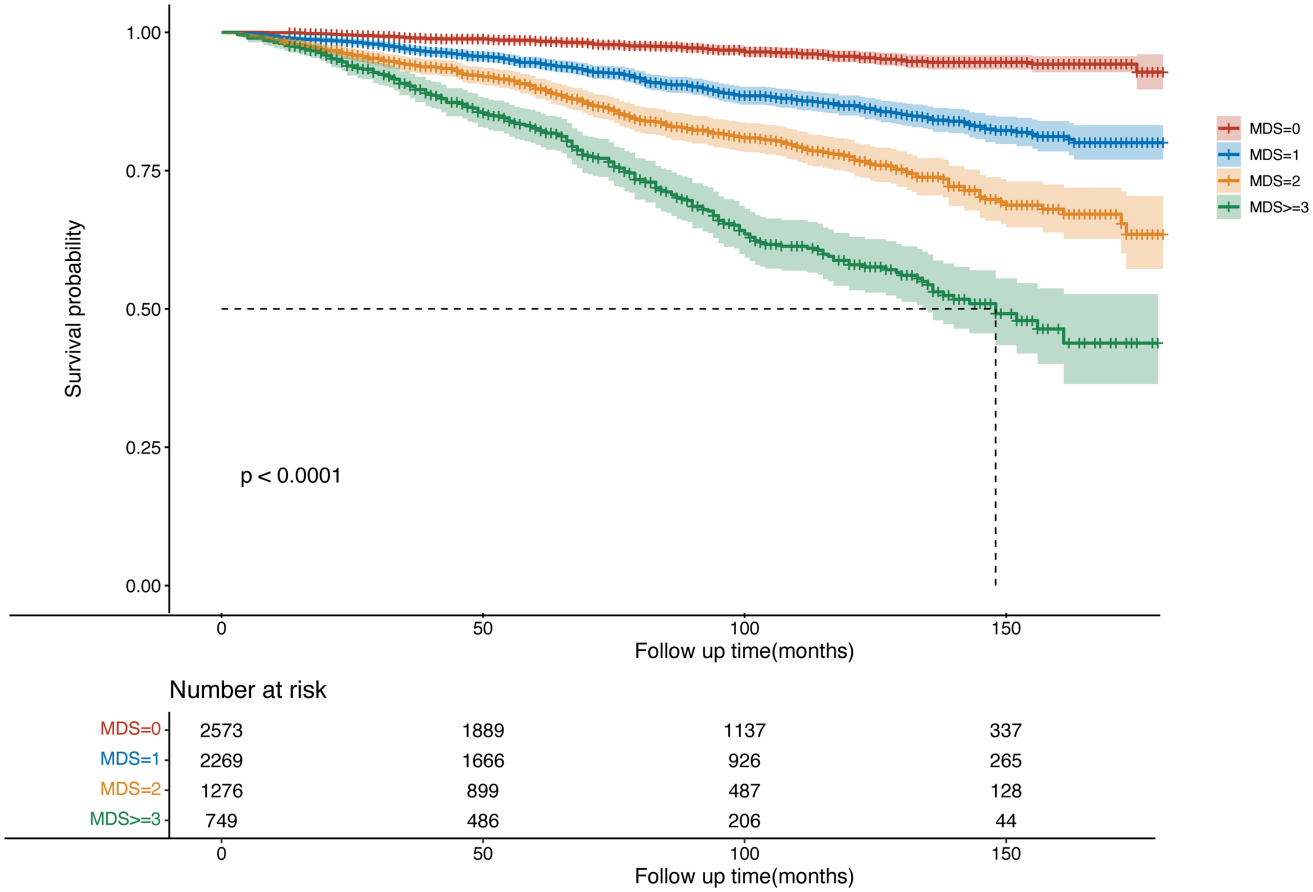
Kaplan–Meier curves were used to present the relationship of the magnesium depletion score with all-cause mortality among participants with UI.

In the UI population, the median follow-up time was 92 months. The median survival time was 148 months in the MDS ≥ 3 group, while the remaining groups did not reach the median survival time. Among 6,867 individuals, 767 deaths were recorded. Weighted Cox regression analysis was used to assess the link between MDS and all-cause mortality (Table 5). The unadjusted model revealed that for each one-unit increase in MDS, the risk of all-cause mortality rose by 1.03 times (OR = 2.03, 95% CI = 1.91–2.17). As different models were applied, the OR decreased, reaching 1.27 in the fully adjusted model. When MDS was treated as a categorical variable with MDS = 0 as the reference group, the unadjusted model indicated OR of 2.86 (95% CI: 1.96–4.16), 5.45 (95% CI: 3.84–7.74), and 12.93 (95% CI: 9.03–18.5) for the MDS = 1, MDS = 2, and MDS ≥ 3 groups, respectively. These OR weakened after adjusting for different models. The results consistently show a positive association between MDS score and the risk of all-cause mortality across various adjustment models. The *p*-value for trend was <0.0001 in all models, indicating a significant upward trend in all-cause mortality with increasing MDS scores. Figure 3 presents the survival outcomes of individuals with UI based on different MDS levels, indicating that as the MDS score increases, the prognosis deteriorates.

**Table 5:** The association between MDS and mortality.

All cause death	Unadjusted model	Model 1	Model 2	Model 3
MDS continue	2.03 (1.91, 2.17)	1.45 (1.34, 1.56)	1.34 (1.24, 1.45)	1.27 (1.13, 1.41)
MDS category				
MDS = 0	ref	ref	ref	ref
MDS = 1	2.86 (1.96, 4.16)	1.70 (1.16, 2.49)	1.69 (1.16, 2.46)	1.46 (0.99, 2.17)
MDS = 2	5.45 (3.84, 7.74)	2.16 (1.50, 3.11)	2.03 (1.43, 2.90)	1.63 (1.10, 2.43)
MDS ≥ 3	12.93 (9.03, 18.50)	3.71 (2.54, 5.42)	3.01 (2.03, 4.45)	2.30 (1.49, 3.54)
*p* for trend	<0.0001	<0.0001	<0.0001	<0.0001

Unadjusted model: No covariates were adjusted. Model 1: Age, race, marital status, education, PIR, BMI, moderate, and vigorous activity were adjusted. Model 2: Model 1 + smoking status, DM, CVD, cancer, MetS, and hyperlipidemia were adjusted. Model 3: Model 2 + vaginal delivery, hysterectomy, albumin, SII, magnesium intake, energy, creatinine, and uric acid were adjusted.

Additionally, we conducted an analysis of the relationship between MDS and all-cause mortality across various types of UI (see Supplementary Table 3). The results indicate that, in both the UI and non-UI populations, as well as across different UI subgroups, MDS ≥ 1 is positively correlated with all-cause mortality when compared to MDS = 0 (as a reference). Furthermore, the impact of MDS on all-cause mortality appears to increase with higher MDS levels (*p* for trend <0.05) regardless of UI status (*p* for interaction >0.05). Supplementary Table 4 illustrates the relationship between various magnesium intakes and MDS in relation to all-cause mortality, indicating that different levels of magnesium intake do not significantly influence MDS or all-cause mortality (*p* for interaction = 0.93). However, MDS is primarily associated with prognosis, as higher MDS levels correlate with poorer outcomes (*p* for trend <0.05). Supplementary Table 5 presents the association of varying magnesium intakes on all-cause mortality in both UI and non-UI populations. The findings suggest that, within the UI population, different magnesium intake levels do not significantly impact prognosis. Conversely, in the non-UI population, higher magnesium intake levels are linked to better prognoses, with this trend being statistically significant (*p* for trend <0.05).

## Discussion

In our study, we uncovered a novel link between magnesium deficiency and UI. Our results showed a positive association between higher MDS scores and the probability of UI. Additionally, individuals with UI and higher MDS scores exhibited a heightened risk of all-cause mortality. Subgroup analyses consistently supported these associations across different population subgroups.

Abnormal levels of magnesium in the body are linked to various diseases, including atherosclerosis, dyslipidemia, abnormal blood glucose, type 2 diabetes, myocardial infarction, hypertension, renal stones, premenstrual syndrome, and mental disorders (29–36). Previous no literature exists on the connection between magnesium deficiency and UI. Studies (19–22) suggest that magnesium supplementation may help alleviate bladder discomfort in postoperative patients and improve symptoms in women with urgency or detrusor instability. Our research found higher magnesium depletion scores in individuals with UI, with more severe symptoms as magnesium levels decreased, underscoring the importance of magnesium deficiency in UI. Additionally, the impact of the MDS on UI varies among different subgroups, such as SUI, where interactions with age, BMI, number of vaginal deliveries, and hypertension were observed. Further studies are needed to investigate the underlying mechanisms behind these subgroup differences.

Magnesium deficiency may impair bladder function through dysregulation of smooth muscle activity. For example, in a randomized trial by Gordon et al. (22), 55% women with detrusor instability receiving magnesium hydroxide experienced a subjective improvement in urinary urgency. Similarly, preclinical studies demonstrate that magnesium depletion increases bladder smooth muscle contractility via elevated intracellular calcium influx (18, 42, 43). These findings, combined with magnesium’s known role in calcium channel regulation, provide multilevel support for our hypothesis.

Lower serum magnesium levels have been linked to increased mortality risk in various diseases (44–47), including cardiovascular mortality, acute myocardial infarction mortality, and chronic kidney disease mortality. This study is the first to identify a higher risk of all-cause mortality in UI patients with elevated MDS, with mortality risk rising with the severity of magnesium deficiency. Previous research (23) demonstrated that low magnesium intake and elevated MDS levels significantly influence the risk of all-cause mortality in a large sample population. In the cohort participating in the UI survey of this study, our findings indicate that the risk of all-cause mortality associated with MDS is primarily linked to the level of MDS itself, independent of UI status and magnesium intake levels. Notably, in the non-UI population, higher magnesium intake correlates with improved prognosis. Additionally, research by Ferre et al. (48) found that MDS is not associated with the presence of CKD, but may be related to serum magnesium levels. Our study further suggests that the impact of MDS on all-cause mortality remains independent of UI status. The findings of this research enhances our comprehension of the significance of magnesium status in health and disease, particularly in conditions like UI that have received less attention. Timely interventions could help reduce mortality risk, especially among those with elevated MDS.

This research highlights numerous advantages. It is the initial examination into the link between magnesium deficiency and UI, utilizing a large representative sample of the U.S. population with data collected over seven time periods. The ample sample size ensures robust statistical power for thorough analysis. The use of sample weights enhances the generalizability of the national results. The researchers considered various potential confounders, drawing on prior research and clinical expertise to improve the reliability of the outcomes. The study utilized the MDS as an indicator, offering a more precise reflection of magnesium’s physiological status. Sensitivity analyses were performed to confirm the strength of the results. However, there are some limitations to consider. The identification of participants with UUI and SUI relied on questionnaire surveys in the NHANES database, which may introduce misclassification. The cross-sectional nature of the data hinders establishing a causal relationship between MDS and UI. Despite efforts to control for confounders, the impact of unmeasured factors remains a concern. The absence of serum magnesium data in NHANES limits comparisons with MDS, necessitating further prospective research. Generalizing the results beyond the U.S. population should be done cautiously, as the findings may be specific to this demographic.

## Conclusion

Our study reveals a significant positive association between MDS and the prevalence of UI. Furthermore, elevated MDS levels are linked to an increased risk of all-cause mortality among patients suffering from UI. These findings underscore the importance of monitoring magnesium status in individuals with UI, suggesting that addressing magnesium deficiency may not only improve urinary health but also potentially reduce mortality risks. Consequently, further research is warranted to explore the underlying mechanisms of this relationship and to evaluate the benefits of magnesium supplementation in this vulnerable population.

## Data Availability

Publicly available datasets were analyzed in this study. This data can be found at: https://www.cdc.gov/nchs/nhanes/?CDC_AAref_Val=https://www.cdc.gov/nchs/nhanes/index.htm.

## References

[1] Aoki Y Brown HW Brubaker L Cornu JN Daly JO Cartwright R. Urinary incontinence in women. Nat Rev Dis Primers. (2017) 3:17097. doi: 10.1038/nrdp.2017.97 29143807

[2] Vaughan CP Markland AD. Urinary incontinence in women. Ann Intern Med. (2020) 172:ITC17–32. doi: 10.7326/AITC202002040 32016335

[3] Lukacz ES Santiago-Lastra Y Albo ME Brubaker L. Urinary incontinence in women: a review. JAMA. (2017) 318:1592–604. doi: 10.1001/jama.2017.12137, PMID: 29067433

[4] Milsom I Coyne KS Nicholson S Kvasz M Chen CI Wein AJ. Global prevalence and economic burden of urgency urinary incontinence: a systematic review. Eur Urol. (2014) 65:79–95. doi: 10.1016/j.eururo.2013.08.031, PMID: 24007713

[5] Hu JC Ding YQ Pang HY Yu CQ Sun DJY Pei P. Prevalence of urinary incontinence in middle-aged and elderly adults in 10 areas in China. Zhonghua Liu Xing Bing Xue Za Zhi. (2024) 45:11–8. doi: 10.3760/cma.j.cn112338-20230910-00144, PMID: 38228519

[6] Kannan P Bello UM. Efficacy of various forms of acupuncture for the treatment of urinary incontinence in women: a systematic review and meta-analysis. Explore. (2023) 19:26–35. doi: 10.1016/j.explore.2022.07.004, PMID: 35868972

[7] Trowbridge ER Hoover EF. Evaluation and treatment of urinary incontinence in women. Gastroenterol Clin N Am. (2022) 51:157–75. doi: 10.1016/j.gtc.2021.10.010 35135660

[8] Gallego-Gómez C Rodríguez-Gutiérrez E Torres-Costoso A Martínez-Vizcaíno V Martínez-Bustelo S Quezada-Bascuñán CA. Urinary incontinence increases risk of postpartum depression: systematic review and meta-analysis. Am J Obstet Gynecol. (2024) 231:296–307.e11. doi: 10.1016/j.ajog.2024.02.307 38437894

[9] Yılmaz Bulut T Altay B. Sleep quality and quality of life in older women with urinary incontinence residing in Turkey: a cross-sectional survey. J Wound Ostomy Continence Nurs. (2020) 47:166–71. doi: 10.1097/WON.0000000000000615, PMID: 31913876

[10] Farage MA. Progress in urogenital health: sensitive skin and urinary incontinence. Gynecol Obstet Fertil. (2010) 38:5–8. doi: 10.1016/S1297-9589(10)70014-X, PMID: 20965428

[11] Arroyo-Huidobro M de la Fuente JL Pagespetit MR Perez OM Morera JR López AMA. Incidence of urinary incontinence after hip fracture surgery and associated risk factors: a prospective study. BMC Geriatr. (2024) 24:3. doi: 10.1186/s12877-023-04597-4, PMID: 38166670 PMC10763427

[12] Fwu CW Schulman IH Lawrence JM Kimmel PL Eggers P Norton J. Association of Obesity, metabolic syndrome, and diabetes with urinary incontinence and chronic kidney disease: analysis of the National Health and Nutrition Examination Survey, 2003–2020. J Urol. (2024) 211:124–33. doi: 10.1097/JU.0000000000003761, PMID: 37862455

[13] Yang C Feng Z Chen Z Xu D Li Y Lai K. The risk factors for urinary incontinence in female adults with chronic cough. BMC Pulm Med. (2022) 22:276. doi: 10.1186/s12890-022-02069-w, PMID: 35850673 PMC9295435

[14] Scime NV Hetherington E Metcalfe A Chaput KH Dumanski SM Seow CH. Association between chronic conditions and urinary incontinence in females: a cross-sectional study using national survey data. CMAJ Open. (2022) 10:E296–e303. doi: 10.9778/cmajo.20210147, PMID: 35383034 PMC9259416

[15] Mauseth SA Skurtveit S Langhammer A Spigset O. Incidence of and factors associated with anticholinergic drug use among Norwegian women with urinary incontinence. Int Urogynecol J. (2018) 29:489–95. doi: 10.1007/s00192-017-3499-9, PMID: 29103164

[16] Yang ST Chang WH Wang PH. The impact of parity and mode of delivery on stress urinary incontinence and pelvic organ prolapse may be different. Am J Obstet Gynecol. (2023) 229:350. doi: 10.1016/j.ajog.2023.04.032 37120054

[17] Newberry SJ Tsuei J Larkin J Motala A Howard K Dunivan G. Managing urinary incontinence for women in primary care: environmental scan (base year). Rand Health Q. (2023) 10:3. PMID: 37333668 10.7249/rhq-10-03-03 PMC10273891

[18] Zhou Y Liu X Huang N Chen Y. Magnesium ion leachables induce a conversion of contractile vascular smooth muscle cells to an inflammatory phenotype. J Biomed Mater Res B. (2019) 107:988–1001. doi: 10.1002/jbm.b.34192, PMID: 30270501

[19] Shim JW Cha S Moon HW Moon YE. Effects of intraoperative magnesium and ketorolac on catheter-related bladder discomfort after transurethral bladder tumor resection: a prospective randomized study. J Clin Med. (2022) 11:6359. doi: 10.3390/jcm11216359, PMID: 36362587 PMC9659173

[20] Park JY Hong JH Kim DH Yu J Hwang JH Kim YK. Magnesium and bladder discomfort after transurethral resection of bladder tumor: a randomized, double-blind, placebo-controlled study. Anesthesiology. (2020) 133:64–77. doi: 10.1097/ALN.0000000000003309, PMID: 32304405

[21] Jiang W Zeng X Zhou X Liao O Ju F Zhao Z. Effect of magnesium sulfate perioperative infusion on postoperative catheter-related bladder discomfort in male patients undergoing laparoscopic radical resection of gastrointestinal cancer: a prospective, randomized and controlled study. BMC Anesthesiol. (2023) 23:396. doi: 10.1186/s12871-023-02346-z 38042781 PMC10693125

[22] Gordon D Groutz A Ascher-Landsberg J Lessing JB David MP Razz O. Double-blind, placebo-controlled study of magnesium hydroxide for treatment of sensory urgency and detrusor instability: preliminary results. Br J Obstet Gynaecol. (1998) 105:667–9. doi: 10.1111/j.1471-0528.1998.tb10183.x, PMID: 9647159

[23] Ye L Zhang C Duan Q Shao Y Zhou J. Association of magnesium depletion score with cardiovascular disease and its association with longitudinal mortality in patients with cardiovascular disease. J Am Heart Assoc. (2023) 12:e030077. doi: 10.1161/JAHA.123.030077, PMID: 37681518 PMC10547298

[24] Lyu M Liu J Xu X Liu C Qin H Zhang X. Magnesium alleviates aluminum-induced growth inhibition by enhancing antioxidant enzyme activity and carbon-nitrogen metabolism in apple seedlings. Ecotoxicol Environ Saf. (2023) 249:114421. doi: 10.1016/j.ecoenv.2022.114421, PMID: 36529044

[25] Ponnusamy T Velusamy P Kumar A Morris D Zhang X Ning G. Mitochondrial magnesium is the cationic rheostat for MCU-mediated mitochondrial Ca^2+^ uptake. Res Sq. (2023). doi: 10.21203/rs.3.rs-3088175/v1

[26] Dominguez LJ Veronese N Barbagallo M. Magnesium and the hallmarks of aging. Nutrients. (2024) 16:496. doi: 10.3390/nu16040496, PMID: 38398820 PMC10892939

[27] Simşek E Karabay M Kocabay K. Assessment of magnesium status in newly diagnosed diabetic children: measurement of erythrocyte magnesium level and magnesium tolerance testing. Turk J Pediatr. (2005) 47:132–7. 16052852

[28] Fan L Zhu X Rosanoff A Costello RB Yu C Ness R. Magnesium depletion score (MDS) predicts risk of systemic inflammation and cardiovascular mortality among US adults. J Nutr. (2021) 151:2226–35. doi: 10.1093/jn/nxab138, PMID: 34038556 PMC8349125

[29] Kostov K Halacheva L. Role of magnesium deficiency in promoting atherosclerosis, endothelial dysfunction, and arterial stiffening as risk factors for hypertension. Int J Mol Sci. (2018) 19:1724. doi: 10.3390/ijms19061724, PMID: 29891771 PMC6032400

[30] Simental-Mendía LE Simental-Mendía M Sahebkar A Rodríguez-Morán M Guerrero-Romero F. Effect of magnesium supplementation on lipid profile: a systematic review and meta-analysis of randomized controlled trials. Eur J Clin Pharmacol. (2017) 73:525–36. doi: 10.1007/s00228-017-2212-8, PMID: 28180945

[31] Simental-Mendía LE Sahebkar A Rodríguez-Morán M Guerrero-Romero F. A systematic review and meta-analysis of randomized controlled trials on the effects of magnesium supplementation on insulin sensitivity and glucose control. Pharmacol Res. (2016) 111:272–82. doi: 10.1016/j.phrs.2016.06.019, PMID: 27329332

[32] Ren Q Wang H Zeng Y Tan X Cheng X Zhou T. The association between serum magnesium levels and gestational diabetes mellitus: a systematic review and meta-analysis. Biol Trace Elem Res. (2023) 201:5115–25. doi: 10.1007/s12011-023-03591-6, PMID: 36790586

[33] Shechter M Shechter A. Magnesium and myocardial infarction. Clin Calcium. (2005) 15:111–5. PMID: 16272621

[34] AlShanableh Z Ray EC. Magnesium in hypertension: mechanisms and clinical implications. Front Physiol. (2024) 15:1363975. doi: 10.3389/fphys.2024.1363975 38665599 PMC11044701

[35] Wu J Yang Z Wei J Zeng C Wang Y Yang T. Association between serum magnesium and the prevalence of kidney stones: a cross-sectional study. Biol Trace Elem Res. (2020) 195:20–6. doi: 10.1007/s12011-019-01830-3, PMID: 31338801

[36] Botturi A Ciappolino V Delvecchio G Boscutti A Viscardi B Brambilla P. The role and the effect of magnesium in mental disorders: a systematic review. Nutrients. (2020) 12:1661. doi: 10.3390/nu12061661, PMID: 32503201 PMC7352515

[37] Kim MM Kreydin EI. The association of serum testosterone levels and urinary incontinence in women. J Urol. (2018) 199:522–7. doi: 10.1016/j.juro.2017.08.093 28847480

[38] Xie L Yu Z Gao F. The association between recent cannabis use and urinary incontinence in women: a population-based analysis of the NHANES from 2009 to 2018. World J Urol. (2022) 40:3099–105. doi: 10.1007/s00345-022-04193-y 36289107

[39] Expert Panel on Detection, Evaluation, and Treatment of High Blood Cholesterol in Adults. Executive summary of the third report of the National Cholesterol Education Program (NCEP) Expert Panel on Detection, Evaluation, And Treatment of High Blood Cholesterol In Adults (Adult Treatment Panel III). JAMA. (2001) 285:2486–97. doi: 10.1001/jama.285.19.2486 11368702

[40] Grundy SM Brewer HB Jr Cleeman JI Smith SC Jr Lenfant C. Definition of metabolic syndrome: report of the National Heart, Lung, and Blood Institute/American Heart Association conference on scientific issues related to definition. Circulation. (2004) 109:433–8. doi: 10.1161/01.CIR.0000111245.75752.C6, PMID: 14744958

[41] Luo S Liu Z Jiao R Li W Sun J Ma S. The associations of two novel inflammation indexes, systemic immune-inflammation index (SII) and system inflammation response index (SIRI), with periodontitis: evidence from NHANES 2009–2014. Clin Oral Investig. (2024) 28:129. doi: 10.1007/s00784-024-05529-1 38300315

[42] Betul Altinisik H Kirdemir P Altinisik U Gokalp O. Effects of magnesium sulfate on airway smooth muscle contraction in rats. Med Glas. (2016) 13:68–74. doi: 10.17392/862-16, PMID: 27452328

[43] Bai Y Zhang J Xu J Cui L Zhang H Zhang S. Magnesium prevents β-glycerophosphate-induced calcification in rat aortic vascular smooth muscle cells. Biomed Rep. (2015) 3:593–7. doi: 10.3892/br.2015.473, PMID: 26171172 PMC4486886

[44] Angkananard T Anothaisintawee T Eursiriwan S Gorelik O McEvoy M Attia J. The association of serum magnesium and mortality outcomes in heart failure patients: a systematic review and meta-analysis. Medicine. (2016) 95:e5406. doi: 10.1097/MD.0000000000005406 27977579 PMC5268025

[45] Fang X Liang C Li M Montgomery S Fall K Aaseth J. Dose-response relationship between dietary magnesium intake and cardiovascular mortality: a systematic review and dose-based meta-regression analysis of prospective studies. J Trace Elem Med Biol. (2016) 38:64–73. doi: 10.1016/j.jtemb.2016.03.014, PMID: 27053099

[46] Jiang L He P Chen J Liu Y Liu D Qin G. Magnesium levels in drinking water and coronary heart disease mortality risk: a meta-analysis. Nutrients. (2016) 8:5. doi: 10.3390/nu8010005, PMID: 26729158 PMC4728619

[47] Teo KK Yusuf S. Role of magnesium in reducing mortality in acute myocardial infarction. A review of the evidence. Drugs. (1993) 46:347–59. doi: 10.2165/00003495-199346030-00002, PMID: 7693427

[48] Ferre S Li X Adams-Huet B Maalouf NM Sakhaee K Toto RD. Association of serum magnesium with all-cause mortality in patients with and without chronic kidney disease in the Dallas heart study. Nephrol Dial Transplant. (2018) 33:1389–96. doi: 10.1093/ndt/gfx275, PMID: 29077944 PMC6454476

