# Supplementary material for: Magnesium depletion scores as a risk factor for prevalence and mortality rates of urinary incontinence: a national survey analysis
Source: Front Nutr. 2025 Apr 30;12:1439134. doi: 10.3389/fnut.2025.1439134 (PMC12076750; doi:10.3389/fnut.2025.1439134)
Supplement: Supplementary file 1 [file Table_1.DOCX]

Supplementary Material

**Supplement Tables**

Supplement Table1 **Demographic and clinical parameters according to OS（N=6867）**

| **Variable** | Total | Alive | Dead | P value |
| --- | --- | --- | --- | --- |
| Age(years) | 53.94±0.27 | 52.33±0.27 | 70.29±0.56 | < 0.0001 |
| Age, % |  |  |  | < 0.0001 |
| ＜50 years | 39.66 | 42.74 | 8.32 |  |
| ≥50 years | 60.34 | 57.26 | 91.68 |  |
| BMI, % |  |  |  | 0.08 |
| ＜25kg/m2 | 24.67 | 24.48 | 28.62 |  |
| 25~30kg/m2 | 27.54 | 27.76 | 27.47 |  |
| ≥30kg/m2 | 47.08 | 47.75 | 43.90 |  |
| Race, % |  |  |  | < 0.0001 |
| Non-Hispanic White | 74.82 | 74.05 | 82.63 |  |
| Non-Hispanic Black | 8.86 | 8.84 | 9.01 |  |
| Mexican Ameirican | 6.64 | 6.98 | 3.22 |  |
| Other Race | 9.68 | 10.12 | 5.14 |  |
| Marital status, % |  |  |  | < 0.0001 |
| Solitude | 38.09 | 35.88 | 60.81 |  |
| Cohabitation | 61.86 | 64.12 | 39.19 |  |
| Education level, % |  |  |  | < 0.0001 |
| Less than or high school | 37.54 | 35.90 | 54.36 |  |
| Above high school | 62.42 | 64.10 | 45.64 |  |
| PIR, % |  |  |  | < 0.0001 |
| ＜1.3 | 19.27 | 19.34 | 33.05 |  |
| 1.3~3.5 | 34.58 | 35.81 | 47.54 |  |
| ≥3.5 | 40.05 | 44.85 | 19.42 |  |
| Smoking status, % |  |  |  | < 0.0001 |
| Never | 58.95 | 59.99 | 48.70 |  |
| Former | 23.49 | 22.60 | 32.65 |  |
| Current | 17.51 | 17.40 | 18.65 |  |
| Moderate, % |  |  |  | < 0.0001 |
| No | 54.59 | 52.64 | 74.50 |  |
| Yes | 45.40 | 47.36 | 25.50 |  |
| Vigorous, % |  |  |  | < 0.0001 |
| No | 81.71 | 80.25 | 96.50 |  |
| Yes | 18.29 | 19.75 | 3.50 |  |
| Hysterectomy, % |  |  |  | < 0.0001 |
| No | 66.94 | 72.09 | 50.64 |  |
| Yes | 28.51 | 27.91 | 49.36 |  |
| Vaginal delivery, % |  |  |  | < 0.0001 |
| 0 | 14.10 | 16.59 | 8.79 |  |
| 1~3 | 41.73 | 48.16 | 35.22 |  |
| >=3 | 33.02 | 35.24 | 55.99 |  |
| DM, % |  |  |  | < 0.0001 |
| No | 73.23 | 75.52 | 51.90 |  |
| PreDM | 8.25 | 7.94 | 11.59 |  |
| DM | 18.29 | 16.54 | 36.51 |  |
| CVD, % |  |  |  | < 0.0001 |
| No | 89.08 | 91.63 | 63.26 |  |
| Yes | 10.92 | 8.37 | 36.74 |  |
| Hypertension, % |  |  |  | < 0.0001 |
| No | 52.87 | 55.94 | 21.60 |  |
| Yes | 47.13 | 44.06 | 78.40 |  |
| Cancer, % |  |  |  | < 0.0001 |
| No | 84.74 | 86.03 | 72.46 |  |
| Yes | 15.16 | 13.97 | 27.54 |  |
| MetS, % |  |  |  | < 0.0001 |
| No | 58.92 | 60.28 | 46.61 |  |
| Yes | 40.85 | 39.72 | 53.39 |  |
| Hyperlipidemia, % |  |  |  | < 0.0001 |
| No | 23.57 | 24.20 | 17.16 |  |
| Yes | 76.42 | 75.80 | 82.84 |  |
| Albumin(g/L) | 41.39±0.07 | 41.48±0.07 | 40.38±0.17 | < 0.0001 |
| SII | 571.94±6.10 | 561.31± 6.06 | 682.93±18.74 | < 0.0001 |
| Dietary magnesium intake(mg) | 270.25±2.52 | 273.28±2.65 | 239.45±5.67 | < 0.0001 |
| Dietary energy(kcal) | 1849.17±12.50 | 1873.84±13.37 | 1598.49±23.40 | < 0.0001 |
| Creatinine(umol/L) | 71.01±0.33 | 69.45±0.29 | 87.48±1.56 | < 0.0001 |
| Uric acid(umol/L) | 296.22±1.18 | 292.62±1.18 | 334.14±4.98 | < 0.0001 |
| MDS , % |  |  |  | < 0.0001 |
| MDS=0 | 35.06 | 37.45 | 10.68 |  |
| MDS=1 | 35.83 | 36.42 | 29.83 |  |
| MDS=2 | 19.21 | 18.21 | 29.35 |  |
| MDS≥3 | 9.91 | 7.92 | 30.15 |  |
| Survival months | 85.65±1.40 | 87.69±1.53 | 64.93±2.01 | < 0.0001 |

Values are presented as mean +/- SD for continuous variables or as weighted percentages for categorical variables.Abbreviation: BMI=Body mass index, PIR=Poverty to income ratio, DM=Diabetes mellitus, CVD=Cardiovascular disease, MetS=Metabolic syndrome, SII=System immune-inflammation index, SUI=Stress urinary incontinence, UUI=Urgent urinary incontinence, MUI=mix urinary incontinence, MDS=Magnesium Depletion Scores.

Supplement Table 2 Association between MDS and UI after Multiple Imputation.

| Variables | Crude model | Adjusted Model (95%CI) |
| --- | --- | --- |
| SUI |  |  |
| MDS continue | 1.24 (1.2~1.28) | 1.05 (1.01~1.1) |
| MDS category |  |  |
| MDS=0 | ref | ref |
| MDS=1 | 1.55 (1.42~1.69) | 1.13 (1.02~1.25) |
| MDS=2 | 2.38 (2.12~2.66) | 1.25 (1.09~1.45) |
| MDS>=3 | 2.78 (2.39~3.23) | 1.24 (1.01~1.51) |
| p for trend | <0.001 | <0.001 |
| UUI |  |  |
| MDS continue | 1.47 (1.42~1.52) | 1.11 (1.05~1.16) |
| MDS category |  |  |
| MDS=0 | ref | ref |
| MDS=1 | 1.74 (1.59~1.92) | 1.21 (1.09~1.35) |
| MDS=2 | 2.94 (2.62~3.3) | 1.41 (1.21~1.63) |
| MDS>=3 | 3.46 (2.97~4.02) | 1.38 (1.13~1.7) |
| p for trend | <0.001 | <0.001 |
| MUI |  |  |
| MDS continue | 1.52 (1.44~1.61) | 1.1 (1.02~1.19) |
| MDS category |  |  |
| MDS=0 | ref | ref |
| MDS=1 | 1.74 (1.57~1.92) | 1.18 (1.05~1.32) |
| MDS=2 | 2.93 (2.59~3.3) | 1.36 (1.16~1.59) |
| MDS>=3 | 3.45 (2.96~4.03) | 1.34 (1.08~1.66) |
| p for trend | <0.001 | <0.001 |

Unadjusted model: no covariates were adjusted.

Adjusted model: age, race, marital status, education, PIR, BMI, moderate and vigorous activity, smoking status, DM, CVD, hypertension, cancer, MetS, hyperlipidemia, vaginal delivery, hysterectomy, albumin, SII, magnesium intake, energy, creatinine and uric acid were adjusted.

Supplement Table 3 The association between MDS and mortality in different type of UI.

| Subgroup | MDS=0 | MDS=1 | MDS=2 | MDS>=3 | p for trend | P for interaction |
| --- | --- | --- | --- | --- | --- | --- |
|  |  | HR(95%CI) | HR(95%CI) | HR(95%CI) |  |  |
| UI |  |  |  |  |  | 0.66 |
| No | ref | 2.05(1.28,3.26) | 2.18(1.32,3.60) | 3.04(1.85,4.99) | <0.0001 |  |
| Yes | ref | 1.57(1.05,2.35) | 1.84(1.22,2.77) | 2.58(1.66,4.01) | <0.0001 |  |
| SUI |  |  |  |  |  | 0.32 |
| No | ref | 2.13(1.31,3.48) | 2.35(1.36,4.07) | 3.03(1.78,5.13) | <0.0001 |  |
| Yes | ref | 1.46(0.92,2.30) | 1.66(1.01,2.72) | 2.55(1.52,4.28) | <0.001 |  |
| UUI |  |  |  |  |  | 0.18 |
| No | ref | 1.44(0.95,2.19) | 1.82(1.20,2.76) | 2.48(1.64,3.73) | <0.0001 |  |
| Yes | ref | 2.56(1.56,4.18) | 2.45(1.42,4.23) | 3.61(1.89,6.88) | <0.001 |  |
| MUI |  |  |  |  |  | 0.89 |
| No | ref | 2.16(1.31,3.55) | 2.34(1.41,3.90) | 3.23(1.84,5.66) | <0.0001 |  |
| Yes | ref | 2.07(1.13,3.78) | 1.81(0.96,3.41) | 2.75(1.26,6.03) | 0.05 |  |

Weighted Cox regression model adjusted for age, race, marital status, education, PIR, BMI, moderate and vigorous activity were adjusted, smoking status, DM, CVD, cancer, MetS, hyperlipidemia, vaginal delivery, hysterectomy, albumin, SII, magnesium intake, energy, creatinine and uric acid.

Abbreviation: UI=Urinary incontinence, SUI=Stress urinary incontinence, UUI=Urgent urinary incontinence, MUI=Mix urinary incontinence, MDS=Magnesium Depletion Scores.

Supplement Table 4 The association between MDS and mortality in subgroup of magnesium intake.

| Magnesium intake | MDS=0 | MDS=1 | MDS=2 | MDS>=3 | p for trend | p for interaction |
| --- | --- | --- | --- | --- | --- | --- |
|  | HR(95%CI) | HR(95%CI) | HR(95%CI) | HR(95%CI) |  | 0.93 |
| Q1 | ref | 2.02(1.11,3.65) | 2.64(1.30,5.35) | 3.36(1.61,7.02) | <0.001 |  |
| Q2 | ref | 1.85(1.12,3.06) | 1.83(1.16,2.89) | 2.99(1.90,4.71) | <0.0001 |  |
| Q3 | ref | 1.55(0.86,2.80) | 1.66(0.91,3.04) | 2.11(1.10,4.06) | 0.02 |  |
| Q4 | ref | 2.25(1.15,4.41) | 2.63(1.24,5.59) | 3.58(1.56,8.22) | 0.002 |  |

Weighted Cox regression model adjusted for age, race, marital status, education, PIR, BMI, moderate and vigorous activity were adjusted, smoking status, DM, CVD, cancer, MetS, hyperlipidemia, vaginal delivery, hysterectomy, albumin, SII, magnesium intake, energy, creatinine and uric acid.

Abbreviation: MDS=Magnesium Depletion Scores.

Supplement Table 5 The association between magnesium intake and mortality.

| Magnesium intake | UI | No UI |
| --- | --- | --- |
|  | HR(95%CI) | HR(95%CI) |
| Q1 | ref | ref |
| Q2 | 1.30(0.98,1.75) | 0.67(0.47,0.94) |
| Q3 | 1.45(0.92,2.28) | 0.55(0.37,0.80) |
| Q4 | 1.26(0.63,2.54) | 0.31(0.19,0.50) |
| p for trend | 0.22 | <0.0001 |

Weighted Cox regression model adjusted for age, race, marital status, education, PIR, BMI, moderate and vigorous activity were adjusted, smoking status, DM, CVD, cancer, MetS, hyperlipidemia, vaginal delivery, hysterectomy, albumin, SII, magnesium intake, energy, creatinine and uric acid.

.
